# Bacteriemia por *Helicobacter cinaedi* en paciente con colitis ulcerosa: a propósito de un caso

**DOI:** 10.1515/almed-2021-0065

**Published:** 2022-01-27

**Authors:** Kateryna Sidak, Ramón Pérez-Tanoira, Peña Gomez-Herruz

**Affiliations:** Servicio de Análisis Clínicos, Hospital Universitario Príncipe de Asturias, Alcalá de Henares, Madrid, España; Servicio de Microbiología, Hospital Universitario Príncipe de Asturias, Alcalá de Henares, Madrid, España; Departamento de Biomedicina y Biotecnología, Facultad de Medicina, Universidad de Alcalá de Henares, Alcalá de Henares, España

**Keywords:** bacteriemia, *Helicobacter cinaedi*, Maldi-TOF

## Abstract

**Objectivos:**

*Helicobacter cinaedi* es un bacilo gramnegativo espirilar que afecta principalmente a pacientes inmunodeprimidos.

**Caso clínico:**

Varón de 49 años que padece colitis ulcerosa desde 1992 y que acudió a las Urgencias de nuestro hospital por fiebre y dolor testicular. El paciente fue dado de alta con diagnóstico de epididimitis aguda izquierda, con probable transmisión sexual. En las Urgencias se le administró ceftriaxona intravenosa y fue dado de alta con doxiciclina como tratamiento durante 10 días y teniendo buena evolución. Los frascos aerobios de hemocultivos fueron positivos a los tres días de la extracción, en la tinción de Gram se observaron bacilos gramnegativos con morfología espiral en forma de sacacorchos. El análisis realizado tanto del contenido de la botella de hemocultivo como de las colonias crecidas en agar Campylosel incubado en ambiente microaerófilo a 42 °C se identificaron como *H. cinaedi* mediante el sistema Maldi-TOF Biotyper 3.0 (Bruker Diagnostics Inc.).

**Conclusiones:**

El análisis directo de la botella de hemocultivo utilizando el sistema Maldi-TOF permitió identificar la etiología de la bacteriemia, ya que *H. cinaedi* no hubiese crecido en los medios habituales de cultivo. No existe ningún consenso con respecto al tratamiento, pero la combinación de ceftriaxona con doxiciclina puede ser eficaz para el tratamiento de la bacteriemia producida por *H. cinaedi*, la cual se produce debido a la translocación de la bacteria desde el tracto gastrointestinal. Esta bacteriemia va asociada al daño de mucosa intestinal por colitis ulcerosa y tiene lugar principalmente en pacientes inmunodeprimidos.

## Introducción

*Helicobacter cinaedi* es un bacilo gramnegativo con morfología espirilar. La primera vez que se aisló fue en 1984 por Fennel en las muestras rectales de varones homosexuales con proctocolitis [1]. *H. cinaedi* puede causar gastroenteritis, celulitis, meningitis, rash o bacteriemia, en la mayoría de los casos afecta a pacientes inmunodeprimidos, aunque en ocasiones puede afectar a inmunocompetentes [2, 3]. Su reservorio natural es el hámster, aunque también ha sido aislado en materia fecal de perros y gatos [4].

## Caso clínico

En nuestro entorno es un microorganismo que se aísla con poca frecuencia, este es el motivo por el que se presenta este caso de bacteriemia producida por *H. cinaedi* en un varón de 49 años que recibía tratamiento con infliximab y que acudió a las Urgencias de nuestro hospital por fiebre y dolor testicular. El paciente refirió haber mantenido relaciones sexuales de riesgo. En la exploración física se apreció un aumento del tamaño del testículo izquierdo con la cola del epidídimo engrosada y dolorosa sin secreción uretral. En la analítica no se observó leucocitosis pero se detectó una proteína C reactiva alta. La orina mostró piuria y hematuria moderada. En el urocultivo se aislaron más de 100.000 unidades formadoras de colonias/mL de *Escherichia coli*. El paciente padece colitis ulcerosa desde 1992, que requirió ingreso dos veces, la primera vez le trataron con ciclosporina, y la segunda con mercaptopurina. El paciente ingresó en 1997 por un nuevo brote de colitis ulcerosa corticorresistente y en el análisis de las heces se detectó la presencia de *Campylobacter jejuni*. En 2019 tuvo epididimitis aguda derecha y le diagnosticaron sífilis y gonorrea. En esta ocasión, el paciente fue dado de alta con diagnóstico de epididimitis aguda izquierda, de probable transmisión sexual, y 100 mg de doxiciclina cada 12 horas durante 10 días como tratamiento. Antes de que el paciente fuese dado de alta, se le administró una dosis de 250 mg de ceftriaxona intravenosa y se le extrajeron dos parejas de hemocultivos que fueron incubados en Biomerieux Virtuo BacT/ALERT durante tres días hasta que se detectó crecimiento en los frascos aerobios de las dos extracciones. No se detectó crecimiento en los dos frascos anaerobios.

Según Araoka et al. [5], los sistemas automatizados tardan una media de cinco días hasta detectar crecimiento de *H. cinaedi* en los frascos de hemocultivos (entre 2–12 días). Se realizó una tinción de Gram al frotis del medio de hemocultivo y se observaron bacilos gramnegativos con morfología espiral en forma de sacacorchos. Mediante el análisis directo del contenido de los dos frascos aerobios de hemocultivos utilizando el sistema Maldi-TOF Biotyper 3.0 (Bruker Diagnostics Inc.) se detectó la presencia de *H. cinaedi* con score 1.7. Después de sembrar los hemocultivos, no hubo crecimiento en las placas de agar chocolate (Biomerieux) incubadas con una atmósfera de 5% de CO_2_, pero sí que lo hubo en el agar Campylosel (Biomerieux), selectivo para *Campylobacter*, incubado en ambiente microaerófilo mediante el uso de BD GasPack EZ Pouch System a 42 °C. *H. cinaedi* crece a 35 °C, aunque hay casos descritos en la literatura del crecimiento de algunas cepas a 42 °C [5, 6]. El estudio, mediante Maldi-TOF Biotyper 3.0, de las colonias crecidas en el agar Campylosel, confirmó la identificación previa de los hemocultivos de *H. cinaedi*. No se puedo estudiar la sensibilidad antimicrobiana de este microorganismo mediante el método Kirby-Bauer debido a que no hubo crecimiento, en agar Campylosel y condiciones microaerófilas, de la bacteria aislada.

Al contactar con el paciente después de conocer los resultados de los hemocultivos, el paciente ya se encontraba asintomático, después de haber terminado el tratamiento antibiótico domiciliario con doxiciclina.

## Discusión

La visualización de bacilos gramnegativos con morfología espiral en forma de sacacorchos en la tinción de Gram del frotis del medio de hemocultivo positivo y el análisis directo del mismo mediante el sistema Maldi-TOF permitió identificar la etiología de la bacteriemia. Esto no habría sido posible al realizar la siembra del hemocultivo positivo en medios habituales como el agar de chocolate.

No existe un consenso claro con respecto al tratamiento, la mayor información existente es para *Helicobacter pylori*. En general *H. cinaedi* es sensible a carbapenems, aminoglucósidos, tetraciclinas, penicilinas, cefalosporinas y resistente a macrólidos, se ha descrito la aparición de resistencia a quinolonas por mutación en ADN girasa [1]. Existen algunas publicaciones que muestran diferentes alternativas para tratar este tipo de bacteriemias: Bateman et al. [7] utilizaron una combinación de ceftriaxona y doxiciclina durante 2 semanas en un paciente que también padecía un mieloma múltiple, Lacruz et al. [2] emplearon imipenem y gentamicina durante 2 semanas y doxiciclina durante 5 semanas en un enfermo con Hepatitis C y Suzuki et al. [3] trataron un caso con quiste hepático con ampicilina/sulbactam*.*

Aunque es poco frecuente, *H. cinaedi* puede llegar a colonizar el tracto gastrointestinal y su translocación puede llevar al desarrollo de bacteriemia asociada al daño de mucosa intestinal por colitis ulcerosa [5]. Esto tiene lugar principalmente en pacientes inmunodeprimidos.

## Puntos clave

– *H. cinaedi* en la mayoría de los casos afecta a los pacientes inmunodeprimidos.
– *H. cinaedi* es un microorganismo de crecimiento lento y exigente, en nuestro caso creció en el agar de Campylosel en ambiente microaerófilo a 42 °C, lo cual retrasa el diagnóstico.
– La clave para detectar la presencia de *H. cinaedi* es la realización de la tinción de Gram de un hemocultivo, aunque por sus caracteristicas inicialmente puede conducirnos a pensar en *Campylobacter* spp.
–
*H. cinaedi* es un microorganismo que presenta cierta dificultad en el aislamiento e identificación, por eso la utilización de la espectrometría de masas Maldi-TOF es de gran ayuda.
– Aunque no existe un esquema aprobado de tratamiento, en la literatura hay ejemplos de las terapias eficaces, que se mencionaron anteriormente.
– *H. cinaedi*, especie enterohepática de *Helicobacter*, causa infrecuente de gastroenteritis y bacteriemia.
